# Molecular characterization of the uncultivatable hemotropic bacterium *Mycoplasma haemofelis*

**DOI:** 10.1186/1297-9716-42-83

**Published:** 2011-07-12

**Authors:** Emily N Barker, Alistair C Darby, Chris R Helps, Iain R Peters, Kate J Heesom, Christopher J Arthur, Ben Crossett, Margaret A Hughes, Alan D Radford, Séverine Tasker

**Affiliations:** 1 School of Veterinary Sciences, University of Bristol, Langford, BS40 5DU, UK; 2 Centre for Genomic Research, Institute of Integrative Biology, University of Liverpool, Liverpool, L69 7ZB, UK; 3 Proteomics Facility, University of Bristol, Bristol, BS8 1TD, UK; 4 School of Chemistry, University of Bristol, Bristol, BS8 1TS, UK; 5 School of Molecular Bioscience, University of Sydney, Sydney, NSW 2006, Australia; 6 Institute of Infection and Global Health, University of Liverpool, Liverpool, L69 3BX, UK

## Abstract

*Mycoplasma haemofelis *is a pathogenic feline hemoplasma. Despite its importance, little is known about its metabolic pathways or mechanism of pathogenicity due to it being uncultivatable. The recently sequenced *M. haemofelis *str. Langford 1 genome was analysed and compared to those of other available hemoplasma genomes.

Analysis showed that in hemoplasmas genes involved in carbohydrate metabolism are limited to enzymes of the glycolytic pathway, with glucose appearing to be the sole energy source. The majority of the pentose phosphate pathway enzymes that catalyze the de novo synthesis of ribonucleotides were absent, as were cell division protein FtsZ and chaperonins GroEL/ES. Uncharacterized protein paralogs containing putative surface expression motifs, comprised 62% of *M. haemofelis *and 19% of *Mycoplasma suis *genome coverage respectively, the majority of which were present in a small number of unstructured islands. Limited mass spectrometry and immunoblot data matched a number of characterized proteins and uncharacterized paralogs, confirming their expression and immunogenicity in vivo.

These data have allowed further characterization of these important pathogens, including their limited metabolic capabilities, which may contribute to their uncultivatable status. A number of immunogenic proteins, and a potential mechanism for host immune system evasion, have been identified.

## Introduction

The hemotropic mycoplasmas (hemoplasmas) are a group of bacteria that can induce hemolytic anemia in a wide variety of mammals [1]. The feline hemoplasma, *Mycoplasma haemofelis*, and the porcine hemoplasma, *Mycoplasma suis *were reclassified as members of the genus *Mycoplasma *within the Mollicutes class following 16S ribosomal RNA gene phylogenetic analysis [1]. Recently the whole genome sequence of *M. haemofelis *str. Langford 1 was published [2]. This low-passage strain has been shown to induce hemolytic anemia in immunocompetent specific pathogen free (SPF)-derived cats [3,4]. Subsequently the annotated genome sequences of two strains of *M. suis *and a further strain of *M. haemofelis *have also been published [5,6].

To date the hemoplasmas have been uncultivatable in vitro, meaning that sourcing large quantities of purified hemoplasma DNA has been difficult. When hemoplasma shotgun libraries have been screened and compared against mass spectrometry data the lack of whole genome sequence data available for hemoplasmas, and the extensive host protein contamination present in preparations, have limited identification of hemoplasma proteins [7,8].

The aim of this study was to fully describe the genome of *M. haemofelis *str. Langford 1, and to compare it with available hemoplasma genomes.

## Materials and methods

### Sources of *M. haemofelis *DNA and protein

Preparations of *M. haemofelis *str. Langford 1 had been previously purified from blood taken from an experimentally infected SPF-derived cat at a time of high parasitemia [9]. DNA and proteins were purified as previously described [7].

### Genome sequencing, analysis and annotation

Whole shotgun pyrosequencing, genome closure and annotation was performed as described elsewhere [2]. The genome was submitted to the European Molecular Biology Laboratory (EMBL) nucleotide sequence database (accession number FR773153).

### Genome sequence data for comparisons

The genome sequences of *M. suis *str. KI_3806 (FQ790233), *M. suis *str. Illinois (CP002525), and *M. haemofelis *Ohio2 (CP002828) were obtained from GenBank. Artemis v12 was used to organize data and facilitate annotation [10]. Orthologs and paralogs were defined using OrthoMCL [11]. Repeat identification was made using MUMmer [12]. Homology was defined as E ≤ 10^-5^.

### Identification of *M. haemofelis *str. Langford 1 proteins

*Mycoplasma haemofelis *str. Langford 1 proteins were prepared for two-dimensional (2D) sodium dodecyl sulfate polyacrylamide gel electrophoresis (SDS-PAGE) using the 2D-clean up kit (GE Healthcare, Amersham Place, Little Chalfont, UK). Proteins were separated according to their isoelectric points on Immobiline™ DryStrip pH 3-11 NL (GE Healthcare) and then according to mass (Criterion XT 4-12% Bis-Tris Precast Gels; Bio-Rad Laboratories Pty. Ltd., Gladesville, NSW Australia) alongside a molecular weight marker (Unstained Precision Plus Protein™ Standards, Bio-Rad Laboratories Ltd., Hemel Hempstead, UK). Gels were stained using Sypro^® ^Ruby Protein Gel Stain (Invitrogen, Paisley, UK). Selected protein spots were picked for trypsin-digestion and the resulting peptides analyzed by liquid chromatography-tandem mass spectrometry (LC-MSMS; QSTAR Elite Q-TOF; AB Sciex Australia Pty. Ltd., Mt Waverley, Victoria, Australia). In addition, data derived from an earlier study using matrix assisted laser desorption/ionization tandem mass spectrometry (MALDI-MSMS) of immunoreactive protein spots were available [7]. Mass spectrometry (MS) data were analyzed using Mascot (Matrix Science Ltd, London, UK) to search a database of in silico predicted *M. haemofelis *proteins, using the following parameters: error tolerance of 0.2 Da for precursor and product ion masses; 1 missed tryptic cleavage; allowing oxidation of methionine and propionamide modification of cysteine as optional modifications. Confident matches were defined by the Mascot score and statistical significance (*p *< 0.05).

## Results

**Figure 1:**
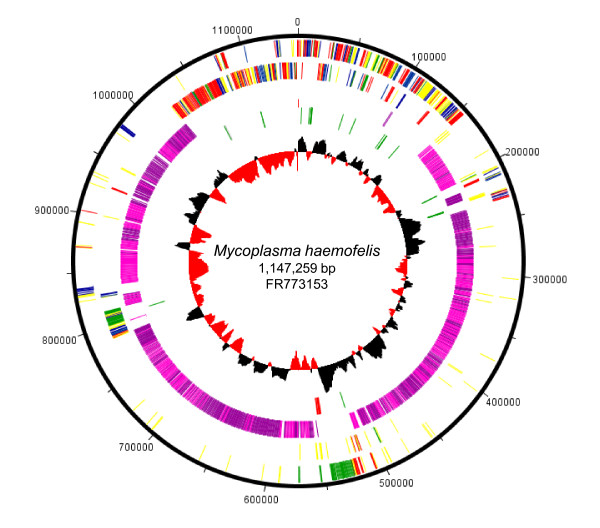
**Circular representation of the *Mycoplasma haemofelis *str. Langford 1 genome**. Outer concentric circle: genomic positions in bases, where position one is the first base of the ***dnaA ***gene. Second concentric circle: the predicted genes on the positive strand (excluding uncharacterized paralog genes). Third concentric circle: the predicted genes on the negative strand (excluding uncharacterized paralog genes). Information storage and processing (red), cellular processes and signaling (green), metabolism (blue), and poorly characterized (yellow). Fourth concentric circle: uncharacterized paralog genes (pink on the forward strand, purple on the negative strand). Fifth concentric circle: tRNA (green), rRNAs (red) and ribonuclease P ribosomal subunit (blue). Sixth concentric circle: the GC-skew diagram; where the black color indicates that the leading strand contains more Gs than Cs, and the red color indicates the more Cs than Gs.

The general features of the *M. haemofelis *str. Langford 1 genome are shown in Figure 1, and these features are compared to other available hemoplasma genomes (*M. haemofelis *str. Ohio2, *M. suis *str. Illinois &*M. suis *str. KI_3806) in Table 1. Both hemoplasmas contained single copies of the 16S, 23S and 5S ribosomal RNA (rRNA) genes, which were located in a single rRNA operon in *M. haemofelis *but not *M. suis*. A single copy of the ribonuclease P β-subunit ribosomal gene was located in each hemoplasma upstream of, and on the complementary strand to, the ATP dependent protease La gene (*lon*). Transfer RNA (tRNA) genes corresponding to each of the amino acids were present in both species, including one assigned to the UGA (opal) codon, consistent with a tryptophan insertion at this position (*M. haemofelis *str. Langford 1 codon usage data: Additional file 1 Table S1). Both *M. suis *strains contained a lysine tRNA complementary to the AAG codon which was absent in *M. haemofelis*, whilst lysine tRNA complementary to the AAA codon was present in both species. From transitions in GC skew, and gene distribution we inferred that the *M. haemofelis *origin of replication is upstream of *dnaA *(HF1_00010) as was also seen for *M. suis*. Using the mycoplasma genetic code Table 1 545 putative open reading frames (ORFs) were identified in the *M. haemofelis *str. Langford 1 with a mean length of 743 nucleotides, of which 328 (21.2% total ORFs) were homologs of proteins from non-hemoplasma bacterial species.

**Table 1:** General attributes of the sequenced hemoplasma genomes.

Hemoplasma species	*Mycoplasma haemofelis* str. Langford 1	*Mycoplasma haemofelis* str. Ohio2	*Mycoplasma suis* str. KI_3806	*Mycoplasma suis* str. Illinois
**EBML accession number**	**FR773153**	**CP002828**	**FQ790233**	**CP002525**

**Topology (chromosome)**	**Circular**	**circular**	**circular**	**circular**

**Length (nucleotides)**	**1 147 259**	**1 155 937**	**709 270**	**742 431**

**G+C content (%)**	**38.9**	**38.8**	**31.1**	**31.1**
1^st ^codon	43.5	43.5	41.0	41.1
2^nd ^codon	37.6	37.6	33.1	33.3
3^rd ^codon	36.0	35.9	20.3	20.1

**Genes per kb**	**1.35**	**1.24**	**1.12**	**1.14**

**Mean coding density % **(mean gene density % i.e. including ribosomal genes)	**94.9 **(95.8)	**94.3 **(95.0)	**87.3 **(90.1)	**89.1 **(90.1)

**Putative ORFs**	**1 545**	**1 548 (inc. 25 pseudo)**	**794 (inc. 15 pseudo)**	**844 (inc. 5 pseudo)**
Uncharacterized ORFs (of which were identified as paralogs)	1 228 (1 115)	1 251 (1,013)	523 (229)	550 (240)

**Structural RNAs**	**35**	**35**	**36**	**36**
Ribosomal RNA (16S; 5S and 23S)	3	3	3	3
Transfer RNA	31	31	32	32
Ribonuclease P β-subunit gene	1	1	1	1

ORF = open reading frame

Of the pentose phosphate pathway enzymes required for *de novo *ribonucleotide synthesis, only the gene encoding ribose phosphate pyrophosphokinase (*prs*) was present in the hemoplasmas; genes encoding transketolase, ribose-5-phosphate isomerase and ribulose-phosphate 3-epimerase were not identified. Alternative purine synthesis pathway homologs inosine-5 monophosphate dehydrogenase (GuaB) and guanosine 3, 5 monophosphate synthetase (GuaA) were however found in both *M. haemofelis *and *M. suis*, whilst homologs for pyrimidine synthesis enzymes cytidylate kinase (*cmk*) and adenosine kinase (HF1_14770) were only found in *M. haemofelis*.

The phosphotransferase system of Mollicutes appears to be present in hemoplasmas, including genes encoding glucose-specific components (*ptsG *&*crr*) and the regulatory protein kinase (*hprK*). Phosphocarrier protein HPr (*ptsH*) of both species contain the His-15 site of enzyme I phosphorylation and the Ser-46 site for HprK phosphorylation. Genes encoding enzymes of the Embden-Meyerhof-Parnas glycolytic pathway catalyzing the conversion of glucose into pyruvate/lactate (Additional file 2 Figure S1) were present in both species whilst genes of the pyruvate dehydrogenase complex were absent, as were genes encoding enzymes of the citric acid cycle. Genes encoding the highly conserved F_O_F_1_-ATP synthase complex (*atpABCDEFGH*) were present to complete the energy pathway, and maintain the transmembrane proton gradient in both species.

Transporters and pathways for the utilization of other energy sources appear to be partial or absent: the glycerol kinase gene (*glpK*) is present whilst the glycerol facilitator gene (*glpF*) is not. Genes required for fructose and mannitol uptake and metabolism (e.g. 1-phosphofructokinase; mannitol-1-phosphate 5-dehydrogenase) were not identified.

Minimal lipid transport and metabolism enzymes were identified. Those that were identified in all three genomes included cardiolipin synthase (*cls*) and choline kinase (*licA*), whilst phosphatidylglycerophosphate synthase (*pgsA*) was truncated in *M. suis *str. KI_3806.

Genome data also suggests that hemoplasmas have minimal amino acid synthesis capabilities, and that they are unable to *de novo *synthesize folate. In contrast, the highly conserved genes encoding the polyamine (spermidine/putrescine) ABC transporter complex (*potABCDE*) were identified in both species, as were coding sequences necessary for the synthesis of all aminoacyl-tRNAs, except glutaminyl-tRNA. In the absence of glutaminyl-tRNA synthetase, genes encoding the aspartyl-tRNA/glutamyl-tRNA amidotransferase (*gatAB*) to convert glutamyl-tRNA into glutaminyl-tRNA were identified in both species.

The ABC phosphate transport system permease genes (*pstAC *&*B*) and other ABC transporter components were also identified including a ferrichrome ABC transporter complex. Predicted proteins with homology to cation transporter proteins including those for cobalt (*cbiO_1_*O_2_*Q*), magnesium (*mgtE*), potassium (*trkAB*) and sodium/calcium were also found.

A gene encoding a DHH family phosphoesterase homologous to *mgpA *was present in both hemoplasma species; however no genes encoding other terminal organelle proteins of the *pneumoniae *group of mycoplasmas were identified. Homologs to *M. suis *proteins HspA1, MSG1 and enolase were predicted in *M. haemofelis *as chaperone protein DnaK, NAD-dependent glyceraldehyde-3-phosphate dehydrogenase and enolase respectively. Genes encoding the components of the chaperonin complex GroEL/ES were not identified in either hemoplasma species, nor was cell-division protein FtsZ.

No predicted *M. haemofelis *ORFs had significant matches to proteins known to specifically mediate colonisation, motility, chemotaxis, natural competence, antibiotic resistance or toxicity in other bacteria, including the P97-like protein of *M. suis *which has a region homologous to *Mycoplasma hyopneumoniae *adhesin P97. In addition to cytidylate kinase and adenosine kinase, the *M. haemofelis *genome also contained genes encoding Mn/Fe- superoxide dismutase (*sodA*), and tRNA modification GTPase (*mnmEg*), which were absent from both *M. suis *strains.

**Figure 2:**
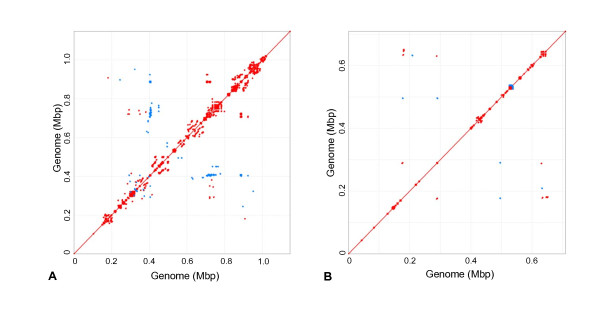
**PROmer dotplot matrix of intra-genomic protein comparisons of Mycoplasma haemofelis str. Langford 1 (A) and Mycoplasma suis. str. KI_3806 (B)**. The genomes were BLASTx compared against themselves. Each dot represents a significant protein match: forward matches are colored red and reverse matches colored blue. Dots present on either side of the diagonal line represent repeated proteins.

Of the 1 228 (79.5% of total ORFs) uncharacterized hypothetical proteins, 1115 (72.2% of total ORFs), covering 61.9% of the *M. haemofelis *str. Langford 1 genome, were highly repeated protein paralogs (Additional file 3 Table S2) mostly contained within three large islands (Figure 1: pink/purple bars). These protein paralogs could be seen as clusters of spots in the in silico predicted proteome of *M. haemofelis *str. Langford 1 (Figure 2a) and *M. haemofelis *str. Ohio2 (data not shown). Similar protein paralogs were detected in both *M. suis *str. KI_3806 (Figure 2b) and *M. suis *str. Illinois (data not shown) and *in silico *predicted proteomes, which contained 550 and 523 uncharacterized hypothetical proteins (including 240 and 229 paralogs; representing 18.9% and 18.5% genome coverage) respectively. Sequence comparisons suggested that they may have arisen by gene duplication events. The predicted motifs of the majority of these putative proteins were consistent with them being expressed on the cell surface; an N-terminal signal peptide or transmembrane region followed by a non-cytoplasmic tail of approximately 200 amino acids. These paralogs were arranged islands flanked by homologs of prolipoproteins, ATP-dependent DNA helicase UvrD/PcrA, C-5 cytosine-specific DNA methylase or genes of the type I restriction-modification system (see Additional file 4 Figure S2 for example of protein paralog "island"). Differences in gene number and genome size between the different strains of hemoplasma species were almost entirely accounted for by differences in the number of genes encoding uncharacterized proteins, mostly those belonging to paralog groups. All characterized genes present in *M. haemofelis *str. Langford 1 were present in *M. haemofelis *str. Ohio 2, and visa versa. Only one characterized gene, type II restriction enzyme DpnII, was present in *M. suis *str. Illinois but not *M. suis *str. KI_3806. Whole genome comparison between strains of *M. haemofelis *identified a 17.4 kb inversion event in a region of the genome containing genes encoding uncharacterized paralogs and other uncharacterized proteins. No large inversion events were detected between strains of *M. suis*.

Nineteen proteins were identified by comparing MS data of selected proteins to in silico predicted ORFs (Additional file 5 Table S3). Of these, seven were matched to characterized proteins, two to conserved hypothetical proteins and the remainder to uncharacterized hypothetical proteins. Of the hypothetical protein matches the strongest (Mascot score of 249; where > 14 was significant) was to hypothetical protein CBY92783, itself located within a string of repeated proteins, contiguous with its sole paralog (CBY92784). Six of the remaining hypothetical protein matches were to members of different protein paralog groups, one of which was known to correspond to an immunogenic protein spot.

## Discussion

The recent publication of the first whole hemoplasma genome sequences [2,5,6] represents a significant step forward in the study of these pathogens. Noticeable differences between the two hemoplasma species include the genome size, 1.15 Mbp for *M. haemofelis *c.f. 0.71-0.74 Mbp for *M. suis*, and G+C content, 38.8-38.9% for *M. haemofelis *c.f. 31.1% for *M. suis*. The differences in genome length can mostly be accounted for by variation in the numbers of the hypothetical proteins, 1 228-1 251 in *M. haemofelis *c.f. 523-550 in *M. suis*, whilst variation at the third codon position of almost 16%, accounts for the majority of the G+C% difference.

*Mycoplasma haemofelis *was also found to have a very low percentage of intergenic regions with ORFs covering 94.9-94.3% of the genome, and ribosomal genes a further 0.9%. In contrast, the mean coding density of *M. suis *strains were 87.3-89.1%, whilst *pneumoniae *group mycoplasmas, to which the hemoplasmas are most closely related based on ribosomal gene phylogeny [13], have mean genome coding densities ranging from 88.3% for *Mycoplasma gallisepticum *(AE015450) to 91.0% for *Mycoplasma genitalium *(L43967).

Mollicutes have previously been reported as bacteria having genomes comprising some of the highest proportions of protein paralogs [14]; for example 22.7% of the genome of plant pathogen "*Candidatus *Phytoplasma asteris" strain OY-M [15] and 15.3% of the genome of porcine respiratory pathogen *M. hyopneumoniae *[16] encode protein paralogs. In comparison, *M. haemofelis *has a dramatically high number of hypothetical protein paralogs representing 61.9-62.7% genome coverage, whilst *M. suis *had only 18.5-18.9% genome coverage by hypothetical protein paralogs. Like in *M. suis *[6] the paralogs of *M. haemofelis *were present mostly within a small number of islands or regions within the genome which did not contain housekeeping genes.

Hemoplasmas experience different environments during the infection cycle: surviving in host tissues and during presumed arthropod vector transmission [17,18]. The small number of sigma factors and transcription regulators identified would suggest a minimal ability to cope with survival outside of the mammalian host, which could be consistent with arthropods only providing a mechanical vector of transmission. The apparent reliance on glucose as the sole carbohydrate energy source would also be consistent with the hemoplasmas being highly adapted to the fairly constant epierythrocytic environment. All cultivatable mycoplasmas for which genome sequence data is available have an intact pentose phosphate pathway for the de novo synthesis of ribonucletoides, whilst the uncultivatable phytoplasmas do not and rely on host cells as a source of ribonucleotides. The absence of an intact pentose phosphate pathway could contribute to the uncultivatable status of the hemoplasmas. However, the presence of genes in *M. haemofelis*, and to a lesser extent in *M. suis*, encoding enzymes that could form alternative synthesis pathways indicate that ribonucleotide metabolism in hemoplasmas requires further investigation, and may not be the same for both species.

The transporter proteins identified indicate that, in addition to glucose, hemoplasmas have the potential to source amino acids, glucose, phosphates and cations from their environment. Mechanisms for the uptake of bases or nucleotides were not identified; however these could be present in the ABC transporters for which substrates were not indentified or in the hypothetical proteins. This finding is similar to other Mollicutes, which are also presumed to source nucleotides from their environment.

Homologs of *sodA*, *rnr *and *mgpA *genes had previously been identified during preliminary screening of the *M. haemofelis *genome as potential virulence factors [19]. Superoxide dismutase activity could represent an important antioxidant defense of *M. haemofelis *to the high oxygen tension in the epierythrocytic environment through catalysis of dismutation of superoxide into oxygen and hydrogen peroxide [20], although its absence from *M. suis *makes this less likely. Genes encoding putative SodA have been reported in phytoplasmas but not other mycoplasmas, in which it is assumed that thioredoxin reductase, which is also present in both hemoplasmas, fulfils the role of superoxide scavenger. Alternatively *M. haemofelis *SodA could act as a virulence factor generating hydrogen peroxide (H_2_O_2_), which damages erythrocytes. Production of reactive-oxygen species, such as H_2_O_2 _by virulent strains of *Mycoides mycoides *subsp. *mycoides *has been suggested as a mechanism for direct toxic effect on host cells [21]. Ribonuclease R has been identified as a pathogenic determinant of both *Shigella flexneri *and *Escherichia coli*, and is required for the expression of a virulent phenotype [22]. However, it has not been reported as a virulence factor in other mycoplasmas and may simply be present in *M. haemofelis *and *M. suis *for its role in messenger RNA degradation, or it may be inactive due to the N-terminal truncation. In the absence of genes encoding the terminal organelle machinery, it is unlikely that *mgpA *will play a role in virulence of hemoplasmas. The absence of these genes also fits with the electron-microscopy studies of *M. haemofelis *and *M. suis *that have not shown evidence of the presence of this "tip"-like structure [23-25]. Also conspicuous in their absence are the highly conserved genes encoding GroEL/ES, FtsZ and the pyruvate dehydrogenase complex, which are also absent in some ureaplasmas and are variably present in other Mollicutes. The significance of their absence is uncertain. The genome of *M. haemofelis *was however shown to contain homologs of all three of the porcine hemoplasma *M. suis *immunodominant proteins: enolase, MSG1 (GapA) and HspA1 (DnaK) [8,26,27], of which MSG1 is thought to play a role in erythrocyte adhesion.

Cyclical parasitemia has been reported as a feature of *M. haemofelis *infection [4], whilst chronic infection is a common feature of hemoplasma infections in general [17]. We suspect that the extensive use of genome space in the metabolically restricted genomes of both hemoplasma species to encode putatively surface expressed paralogs plays a significant role in host immune system evasion, in a similar mechanism to that used by the blood-borne Rickettsial pathogen *Anaplasma marginale *following gene recombination between major super protein genes and their paralogs, which results in antigenic variation [28].

Previous work on the immunoproteome of *M. haemofelis *has indicated that a number of proteins of a variety of molecular weights can stimulate an immune response in the cat [7,9]. Of particular interest is the 25-29 kDa region, which contains a number of immunogenic proteins identified as distinct spots on a 2D immunoblot [7], and as a variable band on one dimensional - polyacrylamide gel electrophoresis [9]. Unfortunately the majority of immunogenic spots in this region did not correspond to visible spots on gels. Further work using the more sensitive LC-MSMS on defined "mass and pI regions" of the gel may be useful in indentifying these proteins, with recombinant expression of selected proteins required to further characterize their role in the immune response. Other options include using narrow range gels with increased loading, a depletion procedure to remove abundant proteins [29], or an immunoaffinity enrichment step [30]. Despite these limitations, in addition to the previously reported phosphoglycerate kinase, chaperone protein DnaK and elongation factor-Ts [7], five novel immunogenic proteins of *M. haemofelis *were identified including one of the uncharacterized protein paralogs. This, and LC-MSMS data matches to other uncharacterized protein paralogs, indicate that not only are hypothetical proteins from these gene duplication events expressed, but that they may also play a role in the host immune response, along with *M. haemofelis *proteins of predicted metabolic function.

In common with other Mollicutes, the genomes of *M. haemofelis *and *M. suis *lack many genes related to amino acid and fatty acid biosynthesis and the citric acid cycle. In the absence of genes encoding pathways for the uptake and utilization of glycerol and fructose, glucose appears to be the sole carbohydrate energy source. It is likely that this has resulted from colonization of the epierythrocytic environment that is rich in glucose, leading to such alternative energy source pathways being rendered redundant. A number of immunogenic proteins have been identified; including metabolic enzymes suspected of having multiple activities within the bacterium, and members of highly repeated families of uncharacterized hypothetical proteins suspected of playing a role in host immune system evasion. Focused MS analysis of purified hemoplasma proteins may allow further characterization of these pathogens.

## Competing interests

The authors declare that they have no competing interests.

## Authors' contributions

IRP and ENB prepared the *M. haemofelis *DNA and protein samples for analysis. MAH, ACD and ENB performed the genome analysis. ENB, KJH, CJA and BC performed the proteome analysis. ENB, ACD, CRH and ST co-drafted the manuscript. ST, CRH and ADR conceived the study, participated in its design and coordination. All authors read and approved the final manuscript.

## Supplementary Material

Additional file 1 **Table S1: Codon usage table for *Mycoplasma haemofelis *str. Langford 1**. Click here for file

Additional file 2 **Figure S1: Predicted energy metabolism pathways of *Mycoplasma haemofelis *str. Langford 1**. Metabolic products are in black. Enzymes are in red, with direction of activity indicated by blue arrows. Click here for file

Additional file 3 **Table S2: Uncharacterised protein paralog families and their corresponding gene numbers**. Click here for file

Additional file 4 **Figure S2: Representative section of the *Mycoplasma haemofelis *str. Langford 1 genome containing a protein paralog "island"**. The "Island" of protein paralog open reading frames (ORFs) is indicated by the pink arrows, with paralog family (PF) number shown. ORFs for poorly characterized proteins are in yellow, including non-repeated hypothetical proteins (HP) and HIT family protein (***hit***). The ORF encoding a membrane lipoprotein is in green and the ORF of metabolic enzyme nicotinate-nucleotide adenylyltransferase (***nadD***) is in blue. Nucleotide position is indicated by numbers and direction of read indicated by arrow head. Click here for file

Additional file 5 **Table S3: Mascot score and gene identity (ID) for protein spots selected for mass spectrometry analysis**. Click here for file
