# Supplementary material for: Molecular characterization of the uncultivatable hemotropic bacterium Mycoplasma haemofelis
Source: Vet Res. 2011 Jul 12;42(1):83. doi: 10.1186/1297-9716-42-83 (PMC3146833; doi:10.1186/1297-9716-42-83)
Supplement: Additional file 1 — Table S1: Codon usage table for Mycoplasma haemofelis str. Langford 1. [file 1297-9716-42-83-S1.DOCX]

## Table S1: Codon usage table for *Mycoplasma haemofelis* str. Langford 1

IUPAC amino acid code, codon sequence, percentage of total encoding codons, ratio per codon per amino acid and total number of occurrences in coding sequence within the genome. *UUG and GUG were used as alternative start codons 111 (7.2 %) and 177 (11.5 %) times respectively

| **Codon** | | **% total** | **Ratio** | **Total** | **Codon** | | **% total** | **Ratio** | **total** | **Codon** | | **% total** | **Ratio** | **total** | **Codon** | | **% total** | **Ratio** | **total** |
| --- | --- | --- | --- | --- | --- | --- | --- | --- | --- | --- | --- | --- | --- | --- | --- | --- | --- | --- | --- |
| F | uuu | 2.2 | 0.57 | 8133 | S | ucu | 3.5 | 0.35 | 12645 | Y | uau | 2.1 | 0.62 | 7483 | C | ugu | 1.0 | 0.66 | 3706 |
| F | uuc | 1.7 | 0.43 | 6205 | S | ucc | 2.1 | 0.21 | 7477 | Y | uac | 1.2 | 0.38 | 4494 | C | ugc | 0.5 | 0.34 | 1879 |
| L | uua | 3.0 | 0.33 | 11052 | S | uca | 1.3 | 0.13 | 4608 |  | uaa | stop | | | W | uga | 1.3 | 0.63 | 4645 |
| L | uug* | 1.6 | 0.17 | 5720 | S | ucg | 0.4 | 0.04 | 1434 |  | uag | stop | | | W | ugg | 0.8 | 0.37 | 2729 |
| L | cuu | 2.0 | 0.22 | 7222 | P | ccu | 1.2 | 0.44 | 4306 | H | cau | 0.9 | 0.63 | 3115 | R | cgu | 0.2 | 0.08 | 887 |
| L | cuc | 0.5 | 0.05 | 1775 | P | ccc | 0.6 | 0.24 | 2303 | H | cac | 0.5 | 0.37 | 1867 | R | cgc | 0.1 | 0.02 | 281 |
| L | cua | 1.4 | 0.15 | 5110 | P | cca | 0.7 | 0.24 | 2385 | Q | caa | 1.9 | 0.64 | 6826 | R | cga | 0.2 | 0.07 | 810 |
| L | cug | 0.6 | 0.07 | 2153 | P | ccg | 0.2 | 0.08 | 771 | Q | cag | 1.0 | 0.36 | 3793 | R | cgg | 0.1 | 0.14 | 323 |
| I | auu | 2.2 | 0.40 | 8049 | T | acu | 2.9 | 0.50 | 10428 | N | aau | 3.8 | 0.74 | 13684 | S | agu | 1.7 | 0.17 | 6257 |
| I | auc | 1.1 | 0.19 | 3864 | T | acc | 1.2 | 0.21 | 4301 | N | aac | 1.3 | 0.26 | 4840 | S | agc | 1.0 | 0.10 | 3481 |
| I | aua | 2.2 | 0.40 | 8078 | T | aca | 1.3 | 0.22 | 4635 | K | aaa | 3.9 | 0.36 | 14119 | R | aga | 1.6 | 0.50 | 5889 |
| M | aug | 1.4 | 1.00 | 4939 | T | acg | 0.4 | 0.07 | 1413 | K | aag | 6.9 | 0.64 | 25071 | R | agg | 1.0 | 0.30 | 3495 |
| V | guu | 2.3 | 0.45 | 8389 | A | gcu | 2.9 | 0.48 | 10560 | D | gau | 4.9 | 0.79 | 17932 | G | ggu | 1.4 | 0.23 | 5246 |
| V | guc | 0.5 | 0.10 | 1831 | A | gcc | 1.2 | 0.20 | 4352 | D | gac | 1.3 | 0.21 | 4659 | G | ggc | 0.6 | 0.10 | 2223 |
| V | gua | 1.6 | 0.30 | 5620 | A | gca | 1.4 | 0.23 | 5108 | E | gaa | 3.8 | 0.51 | 13744 | G | gga | 3.1 | 0.49 | 11334 |
| V | gug* | 0.8 | 0.16 | 2999 | A | gcg | 0.6 | 0.09 | 1996 | E | gag | 3.7 | 0.49 | 13263 | G | ggg | 1.2 | 0.19 | 4486 |
