# Supplementary figures and images for: Molecular characterization of the uncultivatable hemotropic bacterium Mycoplasma haemofelis
Source: Vet Res. 2011 Jul 12;42(1):83. doi: 10.1186/1297-9716-42-83 (PMC3146833; doi:10.1186/1297-9716-42-83)

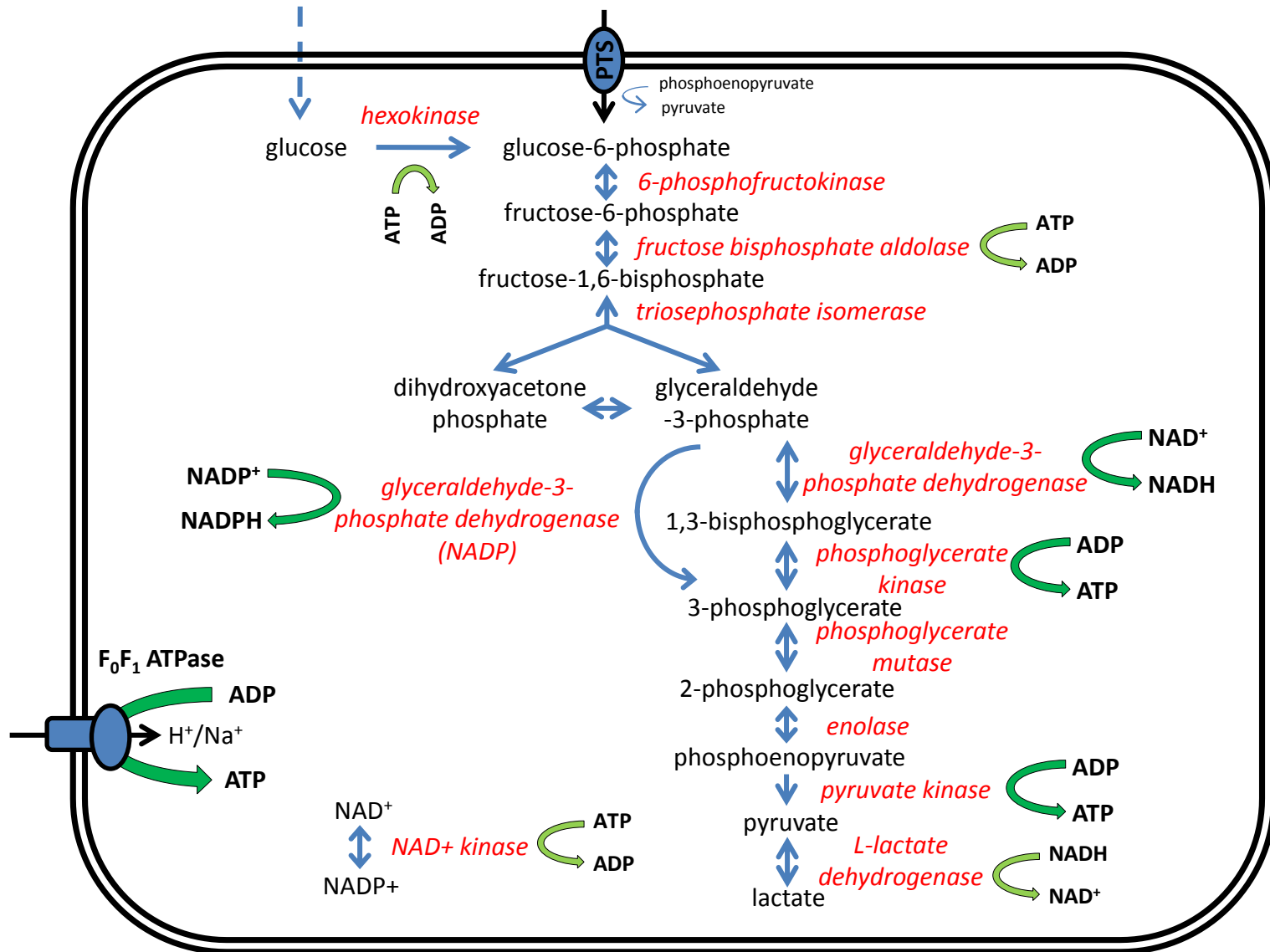

Supplement: Additional file 2 — Figure S1: Predicted energy metabolism pathways of Mycoplasma haemofelis str. Langford 1. Metabolic products are in black. Enzymes are in red, with direction of activity indicated by blue arrows. [file 1297-9716-42-83-S2.PDF]

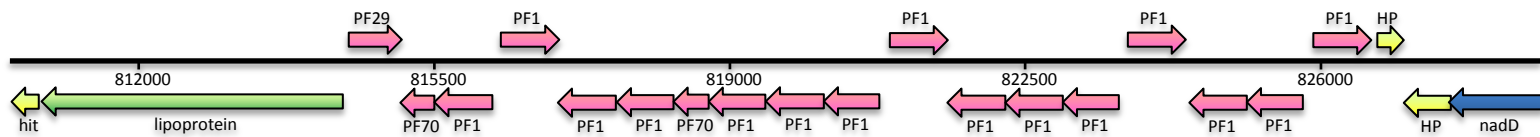

Supplement: Additional file 4 — Figure S2: Representative section of the Mycoplasma haemofelis str. Langford 1 genome containing a protein paralog "island". The "Island" of protein paralog open reading frames (ORFs) is indicated by the pink arrows, with paralog family (PF) number shown. ORFs for poorly characterized proteins are in yellow, including non-repeated hypothetical proteins (HP) and HIT family protein (hit). The ORF encoding a membrane lipoprotein is in green and the ORF of metabolic enzyme nicotinate-nucleotide adenylyltransferase (nadD) is in blue. Nucleotide position is indicated by numbers and direction of read indicated by arrow head. [file 1297-9716-42-83-S4.PDF]
