# Supplementary material for: Molecular characterization of the uncultivatable hemotropic bacterium Mycoplasma haemofelis
Source: Vet Res. 2011 Jul 12;42(1):83. doi: 10.1186/1297-9716-42-83 (PMC3146833; doi:10.1186/1297-9716-42-83)
Supplement: Additional file 3 — Table S2: Uncharacterised protein paralog families and their corresponding gene numbers. [file 1297-9716-42-83-S3.DOCX]

**Table S2: Uncharacterised protein paralog families and their corresponding gene numbers.**

| **Paralog Family** | **Number of ORFs** | **Gene Numbers** |
| --- | --- | --- |
| 1 | 202 | HF1_02430, HF1_02440, HF1_02450, HF1_02460, HF1_02470, HF1_02480, HF1_02490, HF1_02500, HF1_02510, HF1_02520, HF1_02530, HF1_02540, HF1_02550, HF1_02560, HF1_02570, HF1_02580, HF1_02590, HF1_02600, HF1_02610, HF1_02620, HF1_02630, HF1_02640, HF1_02650, HF1_02660, HF1_02670, HF1_02690, HF1_02700, HF1_02710, HF1_02760, HF1_02770, HF1_02780, HF1_02790, HF1_02800, HF1_02810, HF1_02820, HF1_02830, HF1_02840, HF1_02850, HF1_02860, HF1_02870, HF1_02880, HF1_02890, HF1_02900, HF1_02910, HF1_02920, HF1_02930, HF1_02940, HF1_02950, HF1_02970, HF1_02980, HF1_02990, HF1_03010, HF1_03020, HF1_03030, HF1_03040, HF1_03050, HF1_03060, HF1_03070, HF1_03080, HF1_03090, HF1_03100, HF1_03110, HF1_03120, HF1_03130, HF1_03170, HF1_03380, HF1_03420, HF1_03430, HF1_03470, HF1_03510, HF1_03610, HF1_03650, HF1_03690, HF1_03720, HF1_03850, HF1_03890, HF1_03920, HF1_03940, HF1_03950, HF1_03960, HF1_04000, HF1_04010, HF1_04150, HF1_04350, HF1_04360, HF1_04410, HF1_04540, HF1_04550, HF1_04570, HF1_04610, HF1_04620, HF1_04720, HF1_04940, HF1_05410, HF1_06170, HF1_07300, HF1_07540, HF1_07720, HF1_08050, HF1_08200, HF1_08250, HF1_08300, HF1_08310, HF1_08360, HF1_08400, HF1_08470, HF1_08500, HF1_08580, HF1_08610, HF1_08620, HF1_08700, HF1_08750, HF1_08800, HF1_08810, HF1_08950, HF1_09190, HF1_09230, HF1_09240, HF1_09290, HF1_09300, HF1_11060, HF1_11070, HF1_11080, HF1_11090, HF1_11110, HF1_11120, HF1_11130, HF1_11140, HF1_11150, HF1_11160, HF1_11170, HF1_11180, HF1_11190, HF1_11200, HF1_11210, HF1_11340, HF1_11350, HF1_11370, HF1_11380, HF1_11410, HF1_11420, HF1_11440, HF1_11470, HF1_11480, HF1_11500, HF1_11530, HF1_11560, HF1_11610, HF1_11620, HF1_11640, HF1_11650, HF1_11680, HF1_11690, HF1_11700, HF1_11720, HF1_11750, HF1_11760, HF1_11790, HF1_11800, HF1_11810, HF1_11850, HF1_11860, HF1_11870, HF1_11920, HF1_11930, HF1_11940, HF1_11950, HF1_11960, HF1_11970, HF1_11990, HF1_12000, HF1_12020, HF1_12030, HF1_12040, HF1_12270, HF1_12300, HF1_12310, HF1_12330, HF1_12340, HF1_12380, HF1_12390, HF1_12420, HF1_12450, HF1_12480, HF1_12490, HF1_12500, HF1_12510, HF1_12520, HF1_12540, HF1_12550, HF1_12570, HF1_12580, HF1_12600, HF1_12610, HF1_12620, HF1_12630, HF1_12640, HF1_12650, HF1_12660, HF1_12670, HF1_12680, HF1_12700 |
| 2 | 179 | HF1_02720, HF1_03150, HF1_03160, HF1_03190, HF1_03200, HF1_03280, HF1_03290, HF1_03300, HF1_03530, HF1_03670, HF1_03820, HF1_03830, HF1_03870, HF1_03900, HF1_03910, HF1_03980, HF1_04030, HF1_04050, HF1_04070, HF1_04080, HF1_04090, HF1_04100, HF1_04120, HF1_04130, HF1_04160, HF1_04180, HF1_04190, HF1_04200, HF1_04210, HF1_04220, HF1_04260, HF1_04290, HF1_04310, HF1_04320, HF1_04330, HF1_04380, HF1_04390, HF1_04430, HF1_04560, HF1_04580, HF1_04590, HF1_04650, HF1_04660, HF1_04670, HF1_04690, HF1_04700, HF1_04740, HF1_04790, HF1_05140, HF1_05190, HF1_05370, HF1_05390, HF1_05420, HF1_06020, HF1_06030, HF1_07310, HF1_07820, HF1_07950, HF1_08140, HF1_08150, HF1_08160, HF1_08170, HF1_08180, HF1_08190, HF1_08210, HF1_08220, HF1_08230, HF1_08240, HF1_08260, HF1_08480, HF1_08590, HF1_08650, HF1_08660, HF1_08670, HF1_08680, HF1_08690, HF1_08710, HF1_08720, HF1_08730, HF1_08740, HF1_08760, HF1_08960, HF1_08990, HF1_09000, HF1_09010, HF1_09020, HF1_09030, HF1_09040, HF1_09050, HF1_09060, HF1_09070, HF1_09080, HF1_09090, HF1_09100, HF1_09110, HF1_09120, HF1_09130, HF1_09140, HF1_09150, HF1_09160, HF1_09170, HF1_09180, HF1_09670, HF1_09680, HF1_09690, HF1_09700, HF1_09720, HF1_09770, HF1_09900, HF1_09920, HF1_10040, HF1_10060, HF1_10070, HF1_10080, HF1_10100, HF1_11400, HF1_11460, HF1_11520, HF1_11580, HF1_11600, HF1_11670, HF1_11740, HF1_11780, HF1_11830, HF1_11910, HF1_11980, HF1_12220, HF1_12250, HF1_12290, HF1_12470, HF1_12560, HF1_12730, HF1_12770, HF1_12790, HF1_12810, HF1_12830, HF1_12860, HF1_12870, HF1_12890, HF1_12900, HF1_12910, HF1_12930, HF1_12940, HF1_12960, HF1_12970, HF1_12990, HF1_13020, HF1_13030, HF1_13100, HF1_13120, HF1_13150, HF1_13160, HF1_13180, HF1_13210, HF1_13230, HF1_13240, HF1_13250, HF1_13260, HF1_13270, HF1_13280, HF1_13290, HF1_13310, HF1_13320, HF1_13330, HF1_13340, HF1_13350, HF1_13360, HF1_13380, HF1_13390, HF1_13400, HF1_13420, HF1_13430, HF1_13450, HF1_13460, HF1_13480, HF1_13490, HF1_13500, HF1_13600, HF1_14170 |
| 3 | 70 | HF1_03260, HF1_03270, HF1_03520, HF1_03560, HF1_04460, HF1_04840, HF1_04920, HF1_04960, HF1_05010, HF1_05030, HF1_05040, HF1_05320, HF1_05330, HF1_05360, HF1_05810, HF1_05820, HF1_05830, HF1_05890, HF1_05950, HF1_05990, HF1_06000, HF1_07340, HF1_07350, HF1_07360, HF1_07370, HF1_07380, HF1_07390, HF1_07400, HF1_07430, HF1_07560, HF1_07570, HF1_07580, HF1_07590, HF1_07600, HF1_07610, HF1_07620, HF1_07630, HF1_07640, HF1_07650, HF1_07690, HF1_07710, HF1_09320, HF1_09330, HF1_09380, HF1_09420, HF1_09430, HF1_09460, HF1_09490, HF1_09500, HF1_12050, HF1_12690, HF1_13710, HF1_13760, HF1_13780, HF1_13790, HF1_13830, HF1_13850, HF1_13880, HF1_13910, HF1_13920, HF1_13930, HF1_13960, HF1_13970, HF1_13980, HF1_14020, HF1_14030, HF1_14040, HF1_14110, HF1_14130, HF1_14150 |
| 4 | 54 | HF1_03140, HF1_03550, HF1_03570, HF1_03880, HF1_03930, HF1_04060, HF1_04110, HF1_04140, HF1_04340, HF1_04370, HF1_04400, HF1_04440, HF1_04640, HF1_04680, HF1_04710, HF1_04800, HF1_04820, HF1_11360, HF1_11390, HF1_11430, HF1_11450, HF1_11490, HF1_11510, HF1_11540, HF1_11550, HF1_11570, HF1_11590, HF1_11630, HF1_11660, HF1_11710, HF1_11730, HF1_11770, HF1_11820, HF1_12010, HF1_12240, HF1_12280, HF1_12320, HF1_12350, HF1_12460, HF1_12530, HF1_12590, HF1_12800, HF1_12820, HF1_12850, HF1_12920, HF1_12950, HF1_12980, HF1_13110, HF1_13170, HF1_13190, HF1_13220, HF1_13300, HF1_13370, HF1_13470 |
| 5 | 52 | HF1_05300, HF1_05350, HF1_05540, HF1_05610, HF1_05620, HF1_05630, HF1_05640, HF1_05650, HF1_05660, HF1_05670, HF1_05690, HF1_05700, HF1_05720, HF1_05940, HF1_05970, HF1_05980, HF1_06390, HF1_06400, HF1_06410, HF1_06420, HF1_06450, HF1_06500, HF1_06510, HF1_07420, HF1_07670, HF1_08270, HF1_08280, HF1_08770, HF1_08780, HF1_09200, HF1_09210, HF1_09260, HF1_13000, HF1_13010, HF1_13130, HF1_13140, HF1_13570, HF1_13590, HF1_13610, HF1_13620, HF1_13630, HF1_13640, HF1_13650, HF1_13670, HF1_13690, HF1_13700, HF1_13770, HF1_13820, HF1_13870, HF1_13900, HF1_13950, HF1_14010 |
| 6 | 50 | HF1_02960, HF1_03230, HF1_03240, HF1_03250, HF1_03330, HF1_03340, HF1_03350, HF1_03400, HF1_03490, HF1_03630, HF1_03780, HF1_03810, HF1_04270, HF1_04280, HF1_04450, HF1_04770, HF1_04780, HF1_04810, HF1_04830, HF1_05430, HF1_05450, HF1_06120, HF1_07320, HF1_07830, HF1_07840, HF1_07850, HF1_07870, HF1_07940, HF1_07960, HF1_07970, HF1_08030, HF1_08100, HF1_08130, HF1_08380, HF1_08410, HF1_08420, HF1_08430, HF1_08490, HF1_08540, HF1_08600, HF1_08630, HF1_08860, HF1_08890, HF1_08900, HF1_08910, HF1_08970, HF1_09950, HF1_09960, HF1_10130, HF1_10140 |
| 7 | 37 | HF1_03580, HF1_04300, HF1_04500, HF1_04870, HF1_04910, HF1_05310, HF1_05340, HF1_05440, HF1_05460, HF1_05480, HF1_05500, HF1_05960, HF1_06050, HF1_06090, HF1_06110, HF1_06130, HF1_06200, HF1_07440, HF1_07680, HF1_07780, HF1_07880, HF1_07910, HF1_07980, HF1_08010, HF1_08040, HF1_08460, HF1_08510, HF1_08790, HF1_08830, HF1_08930, HF1_09220, HF1_09270, HF1_11840, HF1_13580, HF1_13660, HF1_13730, HF1_13890 |
| 8 | 29 | HF1_05160, HF1_05800, HF1_05840, HF1_09640, HF1_09660, HF1_09870, HF1_09880, HF1_09890, HF1_10000, HF1_10020, HF1_10030, HF1_10200, HF1_10220, HF1_10250, HF1_10280, HF1_10290, HF1_10300, HF1_10320, HF1_10330, HF1_10350, HF1_10370, HF1_10390, HF1_10410, HF1_10420, HF1_10440, HF1_10450, HF1_10480, HF1_10490, HF1_10720 |
| 9 | 21 | HF1_05250, HF1_10010, HF1_10230, HF1_10170, HF1_10260, HF1_10270, HF1_10310, HF1_10340, HF1_10360, HF1_10380, HF1_10400, HF1_10430, HF1_10470, HF1_10630, HF1_10650, HF1_10670, HF1_10700, HF1_10740, HF1_10750, HF1_10780, HF1_10810 |
| 10 | 20 | HF1_01550, HF1_01590, HF1_01600, HF1_01610, HF1_01620, HF1_01630, HF1_01670, HF1_01710, HF1_01720, HF1_01730, HF1_01780, HF1_01850, HF1_01970, HF1_01980, HF1_02000, HF1_02010, HF1_01950, HF1_01580, HF1_01960, HF1_01990 |
| 11 | 20 | HF1_03210, HF1_03310, HF1_04170, HF1_04240, HF1_04750, HF1_05130, HF1_05150, HF1_05210, HF1_07290, HF1_07530, HF1_07810, HF1_09710, HF1_09730, HF1_09760, HF1_09780, HF1_09910, HF1_09930, HF1_10050, HF1_10090, HF1_10110 |
| 12 | 19 | HF1_01510, HF1_01640, HF1_01650, HF1_01680, HF1_01790, HF1_01800, HF1_01810, HF1_02020, HF1_02040, HF1_02050, HF1_02070, HF1_02080, HF1_02090, HF1_07330, HF1_07550, HF1_07800, HF1_08110, HF1_08640, HF1_08980 |
| 13 | 19 | HF1_05100, HF1_05170, HF1_05180, HF1_05200, HF1_09550, HF1_09560, HF1_09580, HF1_09600, HF1_09610, HF1_09620, HF1_09750, HF1_09810, HF1_09830, HF1_12070, HF1_12090, HF1_12110, HF1_12140, HF1_12150, HF1_12720 |
| 14 | 18 | HF1_03220, HF1_03320, HF1_03390, HF1_03480, HF1_03620, HF1_03660, HF1_03770, HF1_03800, HF1_03990, HF1_04230, HF1_04250, HF1_04630, HF1_04760, HF1_06190, HF1_09740, HF1_09790, HF1_09940, HF1_10120 |
| 15 | 18 | HF1_06710, HF1_06720, HF1_09650, HF1_10210, HF1_10240, HF1_10460, HF1_10500, HF1_10510, HF1_10530, HF1_10540, HF1_10560, HF1_10590, HF1_10600, HF1_10660, HF1_10690, HF1_10760, HF1_10790, HF1_10800 |
| 16 | 17 | HF1_01520, HF1_01540, HF1_01660, HF1_01820, HF1_01840, HF1_02030, HF1_02060, HF1_02100, HF1_02130, HF1_02150, HF1_04490, HF1_04860, HF1_04900, HF1_07770, HF1_07890, HF1_08020, HF1_08520 |
| 17 | 17 | HF1_03370, HF1_03410, HF1_03460, HF1_03500, HF1_03540, HF1_03600, HF1_03640, HF1_03790, HF1_05280, HF1_05290, HF1_05870, HF1_05880, HF1_05920, HF1_05930, HF1_08320, HF1_08820, HF1_09250 |
| 18 | 17 | HF1_05080, HF1_05120, HF1_09310, HF1_09350, HF1_09360, HF1_09370, HF1_09400, HF1_09410, HF1_09440, HF1_09450, HF1_09470, HF1_09480, HF1_09510, HF1_09520, HF1_09590, HF1_09840, HF1_12120 |
| 19 | 17 | HF1_05470, HF1_05490, HF1_05520, HF1_05550, HF1_05560, HF1_06150, HF1_06160, HF1_06180, HF1_06220, HF1_06230, HF1_06240, HF1_06600, HF1_06610, HF1_06620, HF1_06640, HF1_06650, HF1_06660 |
| 20 | 12 | HF1_05260, HF1_05270, HF1_10520, HF1_10550, HF1_10570, HF1_10580, HF1_10610, HF1_10620, HF1_10680, HF1_10710, HF1_10730, HF1_10770 |
| 21 | 12 | HF1_05680, HF1_05710, HF1_06300, HF1_06310, HF1_06320, HF1_06330, HF1_06340, HF1_06350, HF1_06360, HF1_06370, HF1_06380, HF1_06480 |
| 22 | 11 | HF1_03680, HF1_03700, HF1_03710, HF1_03730, HF1_03760, HF1_03840, HF1_03970, HF1_05770, HF1_05780, HF1_05790, HF1_05850 |
| 23 | 10 | HF1_04470, HF1_04850, HF1_04880, HF1_06440, HF1_06470, HF1_06530, HF1_07790, HF1_07920, HF1_12710, HF1_13550 |
| 24 | 10 | HF1_05090, HF1_05110, HF1_06730, HF1_09570, HF1_09800, HF1_09820, HF1_09850, HF1_12100, HF1_12130, HF1_12080 |
| 25 | 10 | HF1_13810, HF1_13860, HF1_13940, HF1_13990, HF1_14050, HF1_14060, HF1_14080, HF1_14100, HF1_14120, HF1_14140 |
| 26 | 9 | HF1_01700, HF1_01760, HF1_01770, HF1_12400, HF1_12410, HF1_12430, HF1_12440, HF1_13050, HF1_13680 |
| 27 | 9 | HF1_01390, HF1_01430, HF1_01470, HF1_01480, HF1_01500, HF1_01560, HF1_01860, HF1_01870, HF1_01880 |
| 28 | 9 | HF1_10150, HF1_10160, HF1_10180, HF1_10190, HF1_12060, HF1_05220, HF1_05230, HF1_09980, HF1_09970 |
| 29 | 8 | HF1_05510, HF1_05740, HF1_06140, HF1_06210, HF1_06580, HF1_06630, HF1_07500, HF1_11040 |
| 30 | 7 | HF1_03180, HF1_04420, HF1_04730, HF1_05400, HF1_05580, HF1_06040, HF1_06690 |
| 31 | 6 | HF1_04890, HF1_07990, HF1_08000, HF1_08120, HF1_08570, HF1_08940 |
| 32 | 6 | HF1_05380, HF1_06010, HF1_06060, HF1_06070, HF1_06080, HF1_06100 |
| 33 | 6 | HF1_07860, HF1_08390, HF1_08440, HF1_08870, HF1_08880, HF1_08920 |
| 34 | 5 | HF1_01570, HF1_01920, HF1_07450, HF1_07700, HF1_13560 |
| 35 | 5 | HF1_04950, HF1_04980, HF1_07460, HF1_09340, HF1_09390 |
| 36 | 5 | HF1_07900, HF1_08350, HF1_08530, HF1_08840, HF1_09280 |
| 37 | 5 | HF1_12740, HF1_12750, HF1_13070, HF1_13200, HF1_13410 |
| 38 | 5 | HF1_13720, HF1_13740, HF1_13750, HF1_14070, HF1_14090 |
| 39 | 4 | HF1_01380, HF1_01420, HF1_13800, HF1_13840 |
| 40 | 4 | HF1_01530, HF1_01830, HF1_02110, HF1_02140 |
| 41 | 4 | HF1_05050, HF1_05060, HF1_05750, HF1_05760 |
| 42 | 4 | HF1_07270, HF1_07280, HF1_07510, HF1_07520 |
| 43 | 4 | HF1_08060, HF1_08070, HF1_08080, HF1_08090 |
| 44 | 4 | HF1_08290, HF1_08550 , HF1_08560, HF1_14000 |
| 45 | 4 | HF1_08370, HF1_08330, HF1_08340, HF1_12160 |
| 46 | 3 | HF1_01690, HF1_01750, HF1_13040 |
| 47 | 3 | HF1_03360, HF1_03440, HF1_04480 |
| 48 | 3 | HF1_03590, HF1_04930, HF1_10640 |
| 49 | 3 | HF1_04510, HF1_04520, HF1_04530 |
| 50 | 3 | HF1_05000, HF1_05020, HF1_05730 |
| 51 | 3 | HF1_05530, HF1_05600, HF1_06290 |
| 52 | 3 | HF1_05570, HF1_06250, HF1_06670 |
| 53 | 3 | HF1_12170, HF1_12180, HF1_12190 |
| 54 | 3 | HF1_13060, HF1_13530, HF1_13540 |
| 55 | 2 | HF1_01450, HF1_01460 |
| 56 | 2 | HF1_01490, HF1_01890 |
| 57 | 2 | HF1_01740, HF1_01900 |
| 58 | 2 | HF1_01910, HF1_01940 |
| 59 | 2 | HF1_02420, HF1_02680 |
| 60 | 2 | HF1_02740, HF1_02750 |
| 61 | 2 | HF1_04020, HF1_04040 |
| 62 | 2 | HF1_05590, HF1_06700 |
| 63 | 2 | HF1_06260, HF1_06680 |
| 64 | 2 | HF1_06430, HF1_06460 |
| 65 | 2 | HF1_06490, HF1_06520 |
| 66 | 2 | HF1_07240, HF1_07470 |
| 67 | 2 | HF1_07410, HF1_07660 |
| 68 | 2 | HF1_07750, HF1_07760 |
| 69 | 2 | HF1_09990, HF1_07970 |
| 70 | 2 | HF1_11050, HF1_11100 |
| 71 | 2 | HF1_12230, HF1_12260 |
