# Supplementary material for: Molecular characterization of the uncultivatable hemotropic bacterium Mycoplasma haemofelis
Source: Vet Res. 2011 Jul 12;42(1):83. doi: 10.1186/1297-9716-42-83 (PMC3146833; doi:10.1186/1297-9716-42-83)
Supplement: Additional file 5 — Table S3: Mascot score and gene identity (ID) for protein spots selected for mass spectrometry analysis. [file 1297-9716-42-83-S5.DOCX]

## Table S3: Mascot score and gene identity (ID) for protein spots selected for mass spectrometry analysis.

Results arranged in order of Mascot score, where score is -10*Log(P). *****Unknown hypothetical protein paralogous to repeated sequences. ^‡^Unknown hypothetical protein associated with paralogous locus. MALDI-MSMS = matrix assisted laser desorption / ionization tandem mass spectrometry; LC-MSMS = liquid chromatography-tandem mass spectrometry

| **Identity of Protein Spot** | **Gene ID** | **Mascot Score (matched peptides)** | |
| --- | --- | --- | --- |
|  |  | **MALDI-MSMS** | **LC-MSMS** |
| chaperone protein DnaK | HF1_00650 | 53 (2) | 1371 (25) |
| phosphoglycerate kinase | HF1_02380 | 99 (4) | 662 (13) |
| elongation factor Tu | HF1_15000 | 237 (4) | 645 (11) |
| purine nucleoside phosphorylase | HF1_02250 | 53 (2) | 327 (6) |
| triosephosphate isomerase | HF1_15220 | 57 (1) | 284 (6) |
| unknown hypothetical protein^‡^ | HF1_07750 |  | 249 (8) |
| elongation factor Ts | HF1_14860 | 152 (2) |  |
| unknown hypothetical protein* | HF1_03080 |  | 26 (1) |
| conserved hypothetical protein | HF1_15030 |  | 26 (1) |
| conserved hypothetical protein | HF1_00190 |  | 26 (1) |
| ABC transporter | HF1_15070 |  | 23 (1) |
| unknown hypothetical protein* | HF1_04680 | 22 (1) |  |
| unknown hypothetical protein | HF1_13440 |  | 22 (1) |
| unknown hypothetical protein* | HF1_09980 |  | 20 (1) |
| unknown hypothetical protein | HF1_10920 |  | 19 (2) |
| unknown hypothetical protein* | HF1_08980 |  | 19 (1) |
| unknown hypothetical protein | HF1_14760 | 18 (1) |  |
| unknown hypothetical protein* | HF1_04810 |  | 16 (1) |
| unknown hypothetical protein* | HF1_13070 |  | 16 (2) |
